# Eco‐Friendly and Smart Electrospun Food Packaging Films Based on Polyvinyl Alcohol and *Sumac* Extract: Physicochemical, Mechanical, Antibacterial, and Antioxidant Properties

**DOI:** 10.1002/fsn3.70190

**Published:** 2025-04-22

**Authors:** Aylar Shiri, Ehsan Sadeghi, Khadije Abdolmaleki, Farzad Dabirian, Hooman Shirvani, Mahya Soltani

**Affiliations:** ^1^ Student Research Committee, Department of Food Science and Technology, School of Nutrition Sciences and Food Technology Kermanshah University of Medical Sciences Kermanshah Iran; ^2^ Department of Food Science and Technology, School of Nutrition Sciences and Food Technology, Research Center for Environmental Determinants of Health (RCEDH) Health Institute, Kermanshah University of Medical Sciences Kermanshah Iran; ^3^ Research Center of Oils and Fats Kermanshah University of Medical Sciences Kermanshah Iran; ^4^ Department of Materials and Textile Engineering, Faculty of Engineering Razi University Kermanshah Iran; ^5^ Department of Agronomy and Plant Breeding, Faculty of Agriculture Ilam University Ilam Iran

**Keywords:** electrospun, food packaging films, polyvinyl alcohol, smart packaging, *sumac* extract

## Abstract

With the increasing concern over environmental pollution caused by synthetic packaging, there is a growing demand for sustainable, biodegradable, and functional materials in the food industry. In this study, the antioxidant, antimicrobial, physicochemical, and mechanical properties of electrospun edible films based on *sumac* extract and polyvinyl alcohol were investigated. The films demonstrated a clear colorimetric response to pH changes, shifting from red in acidic to yellow in alkaline conditions, making them suitable for food packaging and freshness monitoring. The film containing 30% sumac extract (P‐SE 30%) exhibited strong antimicrobial activity against *Escherichia coli* (17.01 mm) and *Staphylococcus aureus* (18.02 mm), along with acceptable antioxidant activity (46.32%). The film with 10% *sumac* extract showed the best mechanical strength (0.034 MPa). Moreover, moisture content (4.3%) and water vapor permeability (9.49 g mm/m^2^ Pa) were significantly reduced. Also, the physicochemical properties (SEM, FT‐IR, X‐ray, thickness, Opacity, and mechanical) of electrospun films were improved compared to the control sample. In general, this study demonstrates the potential of electrospun films reinforced with *sumac* extract as a smart food packaging solution for enhancing food safety.

## Graphical abstract

The color of the electrospun edible films prepared with sumac extract changed in response to a shift in pH. The electrospun edible films containing the *sumac* extract showed acceptable antioxidant properties. The antibacterial activity of electrospun edible films containing sumac extract was proven against the investigated bacteria. The morphological and physicochemical characteristics of electrospun edible films were deemed acceptable.

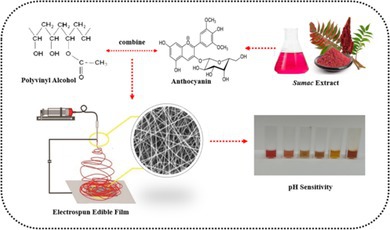

## 1 Introduction

Ensuring that consumers receive safe and high‐quality food is vital, given that factors such as microorganisms, light, moisture, and gases can adversely affect food quality (Azad et al. 2019). Packaging is crucial in the food production process as it protects food from environmental influences and helps reduce waste (Brennan et al. 2021). However, concerns over the environmental impact and the costs associated with recycling plastic packaging have driven food manufacturers to explore biodegradable films (Pelissari et al. 2019).

Food packaging films serve as protective layers that cover food surfaces, making them impermeable to oxygen, odors, oils, and moisture (Shahidi and Hossain 2022). These food packaging films can be made from either natural or synthetic polymers. Polyvinyl alcohol (PVA) is a synthetic, renewable, polar, and water‐soluble polymer that is extensively employed in food packaging due to its desirable mechanical properties, film‐forming capability, chemical resistance, and non‐toxicity (Goudar et al. 2020). This polymer is versatile and has numerous applications, including coating food supplements, protein purification, enzyme stabilization, membrane separation, as well as in the paper and textile sectors (Liu et al. 2020; Maghraby et al. 2023).

In recent years, there has been a growing interest in the food industry in the use of electrospun smart and active food packaging films. These innovative films have the potential to revolutionize food packaging. They are created through electrospinning, which uses high voltage to produce nanoscale fibers from biodegradable polymers. Electrospinning allows for precise control over the mechanical and biological properties of the food packaging films, resulting in materials that are not only biodegradable but also responsive to environmental changes such as pH and temperature (Duan et al. 2023). These food packaging films are embedded with nanoscale sensors capable of releasing antibacterial agents or antioxidants in response to environmental triggers, thus enhancing the shelf life and quality of food products (Topuz and Uyar 2020). Additionally, the biodegradable nature of these food packaging films reduces plastic waste, making them a more environmentally friendly option (Gagaoua et al. 2022; Westlake et al. 2022).

Anthocyanins, known for their potent antioxidant properties, have been identified as valuable components for the development of such food packaging films (Muñoz‐Tebar et al. 2023). These natural pigments, which are non‐toxic and water‐soluble, are responsible for the vibrant red, purple, or blue hues found in various fruits, vegetables, flowers, and grains. Beyond their aesthetic appeal, anthocyanins exhibit antiviral and antimicrobial properties, making them beneficial in food packaging applications (Madivoli et al. 2024a). Their ability to act as highly effective free radical scavengers and to change color under different environmental conditions further enhances their utility in producing biodegradable and smart films (Madivoli et al. 2024b; Mwangi et al. 2024).

*Sumac*, a medicinal plant in traditional medicine, is rich in anthocyanins. The health benefits of *sumac* include fever reduction, support for cardiovascular health, antioxidant effects, antibacterial properties, and anti‐diarrheal activity. The fruit of the *sumac* plant is commonly used as a spice in cooking, characterized by its tart flavor and rich content of anthocyanins, phenolic acids, hydrolysable tannins, terpenoids, citric acid, and malic acid (Hussein et al. 2022; Khoshkharam et al. 2022).

Despite the increasing interest in incorporating anthocyanins into electrospun food packaging films, limited research has focused on the use of sumac‐derived anthocyanins in this context. Previous studies have explored anthocyanin‐based films using extracts from various plant sources, but the unique composition of *Sumac* anthocyanins, along with their strong antioxidant and antimicrobial activity, remains underexplored in electrospun smart films. Additionally, while electrospinning provides a promising technique for developing smart food packaging, further investigation is needed to optimize the integration of sumac anthocyanins to enhance film stability, responsiveness, and functional properties (Stoyanova et al. 2023; Uddin et al. 2024).

Therefore, the objective of this study is to address these research gaps by utilizing sumac‐derived anthocyanins in the production of smart and eco‐friendly food packaging based on polyvinyl alcohol films using the electrospinning technique. This study aims to contribute novel insights into the application of sumac anthocyanins in active food packaging and to evaluate their effectiveness in enhancing the functional properties of biodegradable films.

## 2 Materials and Methods

### 2.1 Materials

PVA (72,000 Da) was sourced from Merck Chemicals (Germany). *Sumac* powder ( *Rhus coriaria* L.) was acquired from local markets in Kermanshah, Iran. 2,2‐Diphenyl‐1‐picrylhydrazyl (DPPH) was purchased from Sigma‐Aldrich Chemical Co. (St. Louis, MO, USA). *Staphylococcus aureus* (ATCC 6538) and *Escherichia coli* (ATCC 25922) were provided by the Microbiology Department, Faculty of Veterinary Medicine, University of Tehran, Iran.

### 2.2 Preparation of Hydroalcoholic Extract of *Sumac*

The hydroalcoholic extract of sumac was obtained using a method similar to that described by Langroodi et al., with some modifications (Langroodi et al. 2019). A total of 350 mL of ethanol and 150 mL of distilled water were mixed with 125 g of *sumac* powder. The mixture was stirred continuously for 24 h. After cooling, the extract was filtered (Whatman No. 1) filter paper. The ethanol and most of the water in the extract were evaporated at 45°C for 1 to 2 h using a rotary evaporator (HAHN SHIN 2005S, KR). The extract was then stored (4°C for 1 to 2 weeks).

### 2.3 Preparation of the Electrospinning Solution

To identify the optimal concentration for the electrospinning solution, PVA concentrations of 5%, 7%, 9%, 11%, and 13% (w/w) were mixed with distilled water. These mixtures were placed in a sealed container with a magnetic stirrer and heated at 45°C for 3 h (Velp Scientifica F20500162, Italy) until the solution was homogeneous. To assess the impact of sumac extract concentration on the characteristics of the films such as color response efficiency, nanofiber morphology, and the mechanical, antioxidant, and antimicrobial properties, sumac extract was incorporated into the optimum PVA concentration (w/w): %10 (P‐SE 10%), %20 (P‐SE 20%), and %30 (P‐SE 30%). These solutions were stirred at 45°C for 3 h. Before preparing the solutions, the *sumac* extract was dried in an oven (Memmert UMB400, DE) at 105°C, and the dry matter content was determined and subtracted from the water content in the electrospinning solution (Rashidi et al. 2021; Zelca et al. 2023).

### 2.4 Electrospinning

The electrospinning setup (ESU) was connected to a 20 kV power supply. A 5 mL needle, attached to a 1 mL plastic syringe filled with the polyvinyl alcohol solution, was linked to the anode. The syringe was mounted horizontally on a digital syringe pump, with the needle directed towards the collector. The needle was connected to the positive electrode of the 20 kV power supply. A positively charged jet of polymer solution formed at the Taylor cone, passed through the air gap, and deposited onto the collector. All electrospinning processes were performed at room temperature in a standard air environment (25°C ± 1°C) with a relative humidity of 45% ± 5%. The electrospinning was conducted in a controlled environment to minimize fluctuations in humidity and temperature, which could impact fiber morphology. Additionally, the airflow in the chamber was maintained at a low level to prevent disturbances in the jet formation and deposition process. Our experimental findings showed that to produce fibers with a smooth, continuous structure and minimal bead formation using the electrospinning technique (Nanoris, Kermanshah, Iran), the flow rate, the distance between the needle tip and the grounded cylindrical collector, the collector speed, and the voltage should be set to 0.1 mL/h, 18 cm, 0.5 rpm, and 20 kV, respectively (Allizond et al. 2023; Łyszczarz et al. 2021).

### 2.5 pH Sensitivity

The pH sensitivity of electrospun nanofiber films was assessed using a modified method previously described by Jiang et al. (2024). Various buffer solutions were created by combining sodium chloride, sodium phosphate, and adjusting the pH with either hydrochloric acid or sodium hydroxide. Nanofiber samples, cut into 1 cm^2^ pieces, were submerged in these buffer solutions with different pH levels (2, 4, 6, 8, 10, and 12) for 1 min. Finally, the color changes that occurred were documented using a camera.

### 2.6 Antimicrobial Activity

Disks of 6–8 mm diameter were cut from the fiber mats and placed on Mueller‐Hinton agar plates that had been inoculated with bacterial strains ( *Escherichia coli* and *Staphylococcus aureus* ). For the assessment, 100 μL of a bacterial culture prepared (105–106 CFU/mL) of the target bacteria was added to 15 mL of Mueller‐Hinton agar. To ensure reliability, both positive and negative controls were included in the experiment. Ciprofloxacin (5 μg/disc) was used as a positive control, while a fiber mat without active compounds served as a negative control. These controls allowed for a comparative evaluation of the antimicrobial effectiveness of the developed electrospun films. The plates were then incubated at 37°C for 24 h, and the inhibition zones were measured with a precise caliper (Duan et al. 2021).

### 2.7 Antioxidant Activity

The DPPH radical scavenging activity of the electrospun nanofibers was measured with slight modifications (Sun et al. 2020). To evaluate the antioxidant activity, 0.02 g of each sample was mixed with 4 mL of ethanol and 1 mL of water, and incubated for 2 h with periodic vertexing. Following this, 1 mL of the sample solution was combined with 4 mL of 0.1 mM methanolic DPPH solution and allowed to react in the dark for 30 min. Absorbance at 517 nm was then measured using a spectrophotometer. A blank was prepared by incubating 1 mL of methanol with 4 mL of 0.1 mM methanolic DPPH in the dark for 30 min. The DPPH scavenging activity was calculated using the following formula:

$$\text{DPPH scavenging activity}\;\left(\%\right)=\frac{\mathrm{Ab}s_{c}-\mathrm{Ab}s_{s}}{\mathrm{Ab}s_{c}}\times 100$$

Abs_c_: Absorbance of the methanolic DPPH solution. Abs_s_: Absorbance of the sample extract solution at 517 nm.

### 2.8 Physicochemical Properties of Electrospun Films

#### 2.8.1 Scanning Electron Microscopy (SEM)

The surface morphology of the nanofibers was examined using SEM (Quanta 450, USA). The samples were coated with a thin layer of gold, and the voltage of the device was 20 kV. At least 40 points of fibers in each photo were randomly selected to check the mean of diameter and frequency range, and these measurements were calculated using ImageJ software (Jiang et al. 2024).

#### 2.8.2 Fourier Transform Infrared (FT‐IR)

FT‐IR spectroscopy was used to identify possible structural interactions between the polymers and indicator dyes. The nanofiber mats were cut into 1 × 1 cm sections and placed in crystal cells, which were then positioned in the FT‐IR spectrometer. This method relies on the absorption of infrared radiation to analyze molecular vibrations and polyatomic ion transitions. The nanofiber spectra were obtained using an FT‐IR spectrophotometer (Irprestige‐21, Shimadzu, Japan) over a wavenumber range of 4000–650 cm^−1^. The spectral data were analyzed to explore the interactions between PVA and sumac extract (Forghani et al. 2022).

#### 2.8.3 X‐ray Diffraction

The X‐ray diffraction pattern of the nanofibers was measured using an X‐ray diffractometer (Philips PW1730, Netherlands) with conditions set at 40 kV, 30 mA, a step size of 1.56056°, and a scanning speed of 1 s per step, using a copper target. The analysis was conducted in a 2*θ* range from 0° to 60° (Jiang et al. 2024).

#### 2.8.4 Mechanical Testing

The mechanical properties of the films were evaluated using a Universal Testing Machine (Zwick, model 1446‐60). Film samples were trimmed to a size of 2 × 7 cm. The distance between the machine's jaws was adjusted to 20 mm, and the speed was set at 50 mm/min. Parameters such as tensile strength (MPa) and elongation at break (%) were determined from the force‐deformation curves (Ahmadi et al. 2020).

#### 2.8.5 Thickness

The thickness of the electrospun films was measured by selecting five points on each film. A digital thickness gauge (Kardo Tech.co, Iran) with an accuracy of 0.001 mm was used to determine the thickness, and the average thickness was calculated in millimeters (Shavisi 2024).

#### 2.8.6 Moisture Content (MC)

To determine the MC, electrospun films were cut into 2 × 2 cm pieces. The samples were weighed before and after drying in an oven at 105°C for 24 h. The MC was calculated using the following formula (Jiang et al. 2024):

$$\mathrm{MC}\;\left(\%\right)=\frac{W_{b}–W_{a}}{W_{a}}\times 100$$

$W_{b}$: Weight of dried sample; $W_{a}:$ Initial weight of sample.

#### 2.8.7 Water Solubility (WS)

To determine solubility, the films were cut into 2 × 2 cm pieces and dried at 105°C for 20 h to obtain the initial dry weight. The films were then submerged in 30 mL of distilled water at 25°C for 20 h. After filtering, the films were dried again (105°C for 20) and weighed. Eventually, solubility was calculated using the following formula (Shavisi 2024).

$$\text{Water Solubility}\;\left(\%\right)=\frac{W_{c}-W_{b}}{W_{c}}\times 100$$

*W*_*c*_: Weight of dried swollen sample; *W*_*b*_: Weight of dried sample.

#### 2.8.8 Water Vapor Permeability (WVP)

The WVP of the nanofibers was determined (ASTM E96‐95 standard method). Permeability cups (an internal diameter of 2 cm) were filled with distilled water to expose the film to 100% relative humidity (RH) inside the cup. The cups were sealed with PVA nanofibers and placed in desiccators containing silica gel. The weight of the cups was recorded every 2 h until a stable weight was achieved. WVP measurements were taken in triplicate (Yildiz et al. 2021):

$$\mathrm{WVP}=\frac{\left(\text{WVTR}\right)\times \left(\Delta x\right)}{\;\left(\;\frac{R_{1}-R_{2}}{100}\right)\times P_{\mathrm{sat}}}$$

WVTR: Rate of water vapor transmission (g m^−2^ s^−1^); *R*_1_: Relative humidity of inside; *R*_2_: Relative humidity outside; *P*_sat_: Saturation vapor pressure of pure water at room temperature; Δ*x*: Thickness of the layer (m).

#### 2.8.9 Optical Properties

The optical characteristics of the electrospun films were assessed using a spectrophotometer (Jenway 6715, UK) to evaluate their ability to block specific wavelengths as per ASTM D1746‐09. The films, cut into 4 × 1 cm pieces, were placed in spectroscopic cells, and their absorbance at 600 nm was recorded. The opacity of the films was calculated using the following formula (Sun et al. 2020):

$$\text{Opacity}=\frac{A600}{T}$$

*T*: Thickness of the samples; A600: Absorbance of the samples at 600 nm.

### 2.9 Statistical Analysis

The experiments were carried out in triplicate (*n* = 3). Statistical evaluation was performed using one‐way ANOVA, and mean comparisons were conducted using Duncan's multiple range test. The analyses were performed with SPSS version 27 (SPSS Inc., Chicago, IL, USA), with a significance level set at 0.05.

## 3 Results and Discussion

### 3.1 pH Sensitivity

**FIGURE 1:**
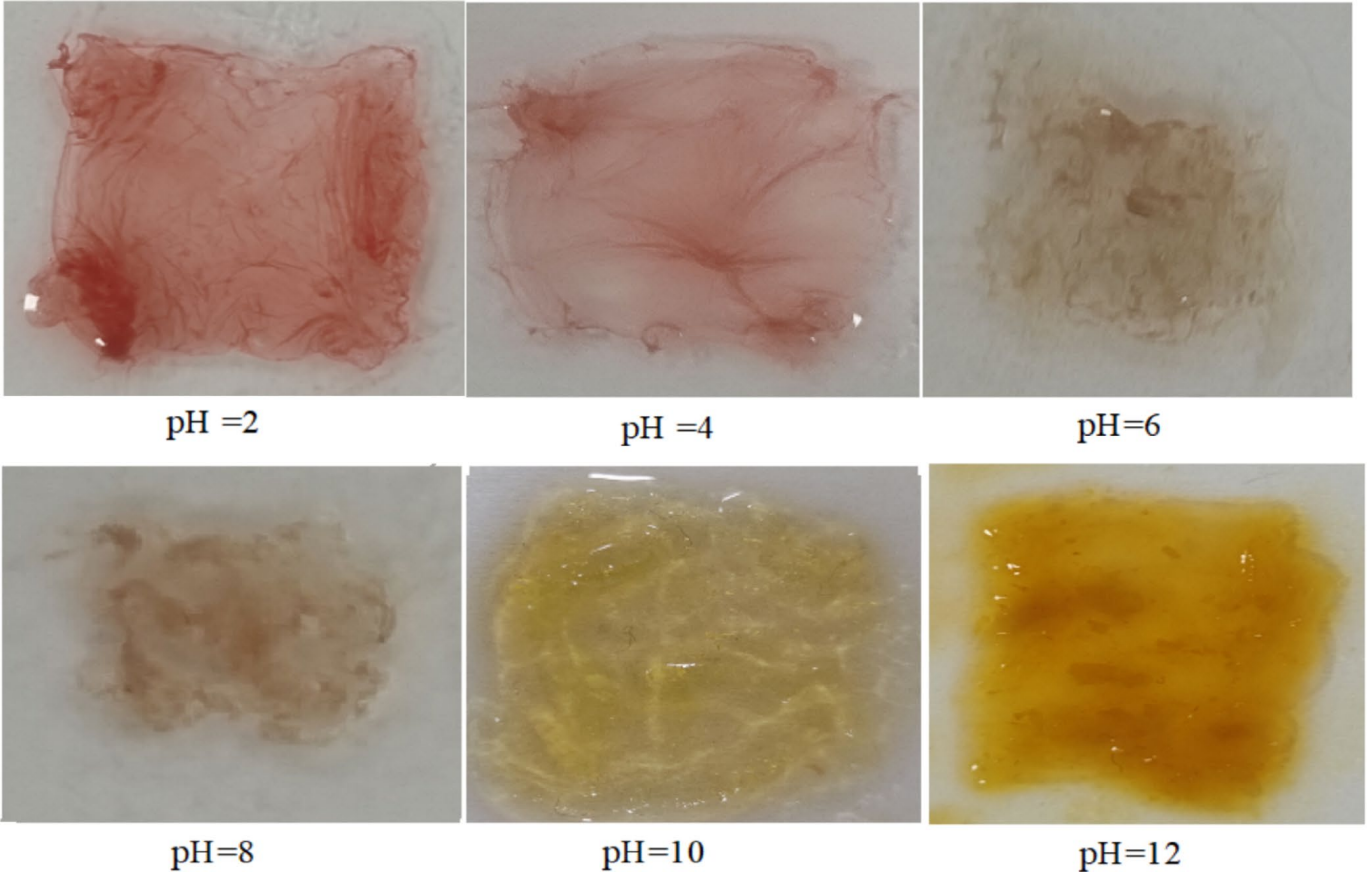
Changed color of electrospun edible films against different pHs.

When food is stored in improper conditions, it spoils and changes its pH. One feature of smart packaging is its sensitivity to pH, which is demonstrated through the color change of the films (Zhao et al. 2022). Anthocyanins undergo structural changes in response to different pH levels. At acidic pHs, they are stable and typically exhibit red or purple colors. However, as the pH approaches neutral or becomes alkaline, their structure changes, leading to color shifts and eventual degradation (Chayavanich et al. 2023). Roy and Rhim (2021) discuss how anthocyanins exhibit pH‐responsive behavior, making them suitable for incorporation into biodegradable polymer‐based films. These pH‐responsive films can provide real‐time assessments of food quality, signaling changes that may indicate spoilage (Roy and Rhim 2021). Figure 1 shows the mat of PVA‐based spun fibers containing sumac extract, which have changed color against different buffers. electrospun films containing concentrations of extract (10%, 20%, and 30%) turned red at pH = 2. As the pH increased to 4, the red color changed to pink. At pH = 6, the pink color changed to brown, and this color remained until pH = 8. A bright yellow color appeared at pH = 10 and, pH = 12 changed the color of films to amber yellow. Therefore, it is possible to consider the electrospun films produced in this research as smart, as they are capable of detecting changes in pH.

In 2022, Farqani et al. conducted a study on electrospun nanofibers and solvent‐cast films based on PVA/κ‐carrageenan containing Centaurea arvensis anthocyanin as a colorimetric pH indicator. The films prepared in their study reacted to the pH change and their color range was from purple to green (Forghani et al. 2022). The ability of anthocyanins to change color in response to pH variations is substantiated by Zhai et al. (2018), who developed a pH‐sensitive film using gelatin, gellan gum, and red radish anthocyanins. Their study demonstrated a distinct color transition from orange‐red to yellow across a pH range of 2–12, illustrating the material's potential as a freshness indicator for smart food packaging. The enhanced mechanical properties and barrier abilities of the film with increased anthocyanin concentration further support its practical application in monitoring food freshness (Zhai et al. 2018). Furthermore, Cao et al. (2019) detailed the development of pH‐sensitive films using purple‐fleshed sweet potato starch and anthocyanins, which also exhibit color changes in response to pH variations. This research further supports the potential of utilizing natural colorants in the creation of smart packaging systems that can effectively monitor food freshness and safety (Cao et al. 2019). The underlying mechanisms of color change in films are critical for their effectiveness as freshness indicators. The incorporation of red cabbage anthocyanins has been shown to create films that monitor food quality through color changes in response to pH variations (Abedi‐Firoozjah et al. 2022). This showcases the potential for developing innovative smart packaging solutions that leverage the natural properties of anthocyanins to enhance food safety.

### 3.2 Antibacterial Activity

Recently, electrospun films have demonstrated significant antibacterial activity, making them promising for food packaging applications. These films are developed using various techniques and materials to enhance their antimicrobial properties (Lopez‐Polo et al. 2023). Electrospun nanofibers have been shown to be loaded with antimicrobial compounds (phenolic compounds, tannins, flavonoids, gallic acid, quercetin and organic acids). These compounds inhibit the growth of various pathogens, including *Escherichia coli* ( *E. coli* ) and *Staphylococcus aureus* ( *S. aureus* ) (AlJuhaimi et al. 2024; Topuz and Uyar 2020). These advancements in electrospun films highlight their potential to improve food safety and extend shelf life by inhibiting microbial growth. *E. coli* and *S. aureus* are significant pathogens in the food industry, posing serious health risks to consumers.

*E. coli* can contaminate food products at various stages, including pre‐harvest, during processing, and in storage environments. Contaminated water supplies used in agriculture can lead to the presence of *E. coli* in fruits and vegetables (Bonten et al. 2021). *S. aureus* can contaminate food through human contact, particularly during food preparation. Hands and utensils used in food handling can be significant sources of contamination (Bencardino et al. 2021). Therefore, these two bacteria were chosen to evaluate the antimicrobial properties of the films. The investigation results of the antibacterial properties show (Table 1) that no antibacterial inhibition zone was observed around the control sample. The antibacterial inhibition zones of P‐SE 10%, P‐SE 20%, and P‐SE 30% samples for *E. coli* were 14 mm, 12 mm, and 17 mm, respectively. Additionally, the inhibition zones for *S. aureus* were reported as 14 mm, 16 mm, and 18 mm. Because anthocyanins have more hydroxyl groups, they are able to inactivate bacteria through hydrogen bonds. In addition, anthocyanin present in sumac extract affects anabolism, catabolism, and bacterial wall (Kauffmann and Castro 2023; Sun et al. 2018).

**TABLE 1:** Antioxidant and antibacterial activity of electrospun edible films composed of polyvinyl alcohol and *sumac* extract.

Sample	DPPH radical scavenging activity (%)	Diameter of inhibition zone (mm)	
		*S. aureus* (+)	*E. coli* (−)
Control	0	0	0
P‐SE 10%	41.04 ± 0.62^b^	14.07 ± 0.11^a^	12.05 ± 0.45^b^
P‐SE 20%	42.98 ± 0.78^b^	16.02 ± 0.02^a^	13.98 ± 0.76^b^
P‐SE 30%	46.32 ± 0.74^a^	18.02 ± 0.03^a^	17.01 ± 0.17^a^
Mean square	21.399*	11.702^ns^	18.754*

*: Significance: All data are shown as mean ± standard deviation (SD). The superscripts different letters in a column indicate significant differences (*P* < 0.05).

The results of the variance analysis (Table 1) indicated that different P‐SE treatments had a statistically significant effect on the inhibition zone diameter against *E. coli* (*p* < 0.05), suggesting a greater enhancement in antimicrobial activity with increasing P‐SE concentration. However, the effect on *S. aureus* was not significant (*p* < 0.05). In this study, P‐SE 30% was reported as a powerful antibacterial agent in the food industry, capable of increasing the shelf life of products. This discrepancy can likely be explained by the structural differences between Gram‐negative and Gram‐positive bacteria. *E. coli* , a Gram‐negative bacterium, possesses an outer membrane that provides a more rigid structure, making it more resistant to antimicrobial agents, including the *Sumac* extract. In contrast, *S. aureus* , a Gram‐positive bacterium, lacks this outer membrane and is therefore more susceptible to the effects of the *Sumac* extract (Huang et al. 2023).

The results of our studies were consistent with those of previous studies. According to the research conducted, researchers found that adding sumac extract to the polymer increased its antibacterial properties. It was also observed that the antimicrobial effectiveness of bioactive compounds found in sumac extract against Gram‐negative bacteria was lower than against Gram‐positive bacteria. Also, in comparison to other bio‐based films, such as those incorporating chitosan or cellulose, Sumac extract‐based films show comparable or superior performance in terms of antimicrobial activity and mechanical properties (Duan et al. 2021).

### 3.3 Evaluation of Antioxidant Potential

Electrospun PVA films can be incorporated with various bioactive compounds such as curcumin, ginger, and chamomile extract to enhance their antioxidant and antimicrobial properties (Köse et al. 2019; Stoyanova et al. 2023; Yang et al. 2021). Phenolic compounds of *sumac extract* have been investigated as factors that cause antioxidant activity in various studies. For example, in a study, researchers used HPLC to investigate the antioxidant and chemical compounds of *sumac*. The results indicated the presence of gallic acid (77.54–389.30 mg/g), flavonoids (2.19–7.54 mg/g), tannins (52.00–189.80 mg/g), and anthocyanins (3.57 to 66.14 mg/g) in Iranian *sumac* (Fereidoonfar et al. 2019). Free radicals are responsible for lipid oxidation and protein degradation in food products, ultimately leading to food spoilage. To prevent this phenomenon and extend the shelf life of food products, antioxidants can be incorporated into packaging materials (Hosseini et al. 2019). Therefore, it was necessary to evaluate the antioxidant capacity of electrospun films. The antioxidant activity of PVA‐based nanofibers containing *sumac* extract was tested through the scavenging of DPPH free radicals. The control sample did not exhibit antioxidant properties; however, incorporating sumac extract at P‐SE 10%, P‐SE 20%, and P‐SE 30% samples significantly increased antioxidant activity to 41.04%, 42.98%, and 46.32%, respectively (Table 1). This effect is attributed to the hydroxyl groups present in sumac, where the hydrogen of the hydroxyl group reacts with DPPH free radicals, resulting in the neutralization of free radicals and reactive oxygen species. Until now, anthocyanins extracted from various plants and fruits have been used in the preparation of films, and their impact on food has been investigated in several studies. A research team produced Kappa‐carrageenan‐polyvinyl alcohol electrospin films combined with anthocyanin and investigated their effect on increasing the shelf life of minced beef meat. Next, the antimicrobial and antioxidant activity of the films was investigated, and the results indicated that these films can increase the shelf life of minced beef meat (Goudarzi et al. 2023). In other studies, the antioxidant activity of films prepared using bioactive compounds found in plant extracts was investigated. Positive results were obtained, showing that increasing the concentration of the plant extract also increased its antioxidant properties (de Barros et al. 2024; Estevez‐Areco et al. 2020; Roshani‐Dehlaghi et al. 2024; Zeinali et al. 2021; Zhao et al. 2023).

### 3.4 Surface Morphology and Microstructural Analysis

The addition of bioactive compounds or other additives can significantly affect the morphology of the electrospun fibers. For instance, when betel leaf ethanol extract was added to electrospun gelatin/chitosan nanofibers on polylactic acid films, SEM imaging revealed the formation of beaded nanofibers at higher concentrations of the extract (Tagrida et al. 2022).

**FIGURE 2:**
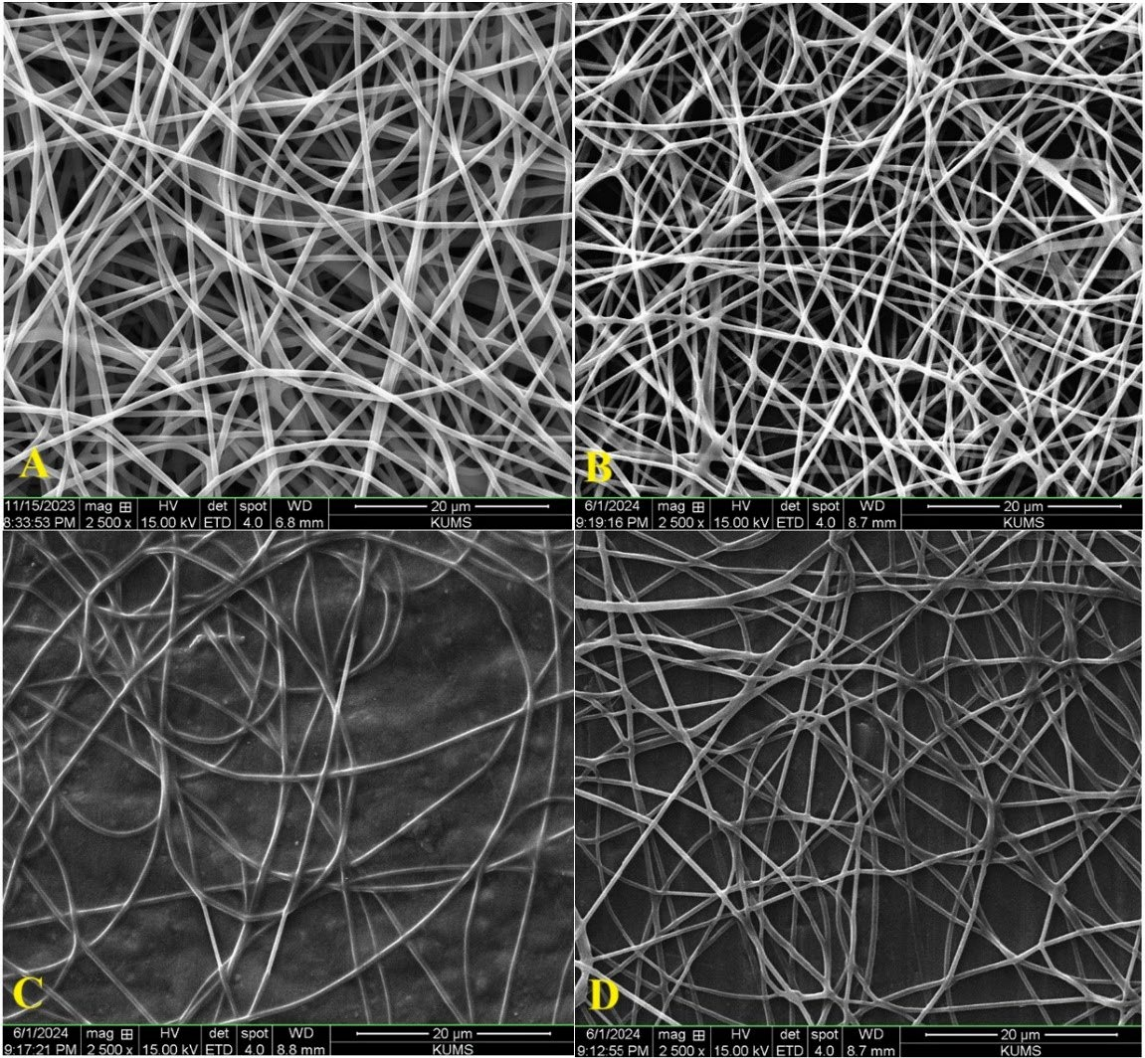
SEM images of the nanofiber structure of electrospun edible films: Control (A), P‐SE 10% (B), P‐SE 20% (C), and P‐SE 30% (D).

Figure 2 displays scanning electron microscope images of the nanofiber structure based on polyvinyl alcohol with varying concentrations of *sumac* extract. Control samples are cylindrical, smooth, thick, and spun without any knots. Additionally, the SEM images of P‐SE 10%, P‐SE 20%, and P‐SE 30% were similar to the control sample in terms of morphology. This similarity indicates that the sumac extract is uniformly distributed within the structure of the nanofibers.

**FIGURE 3:**
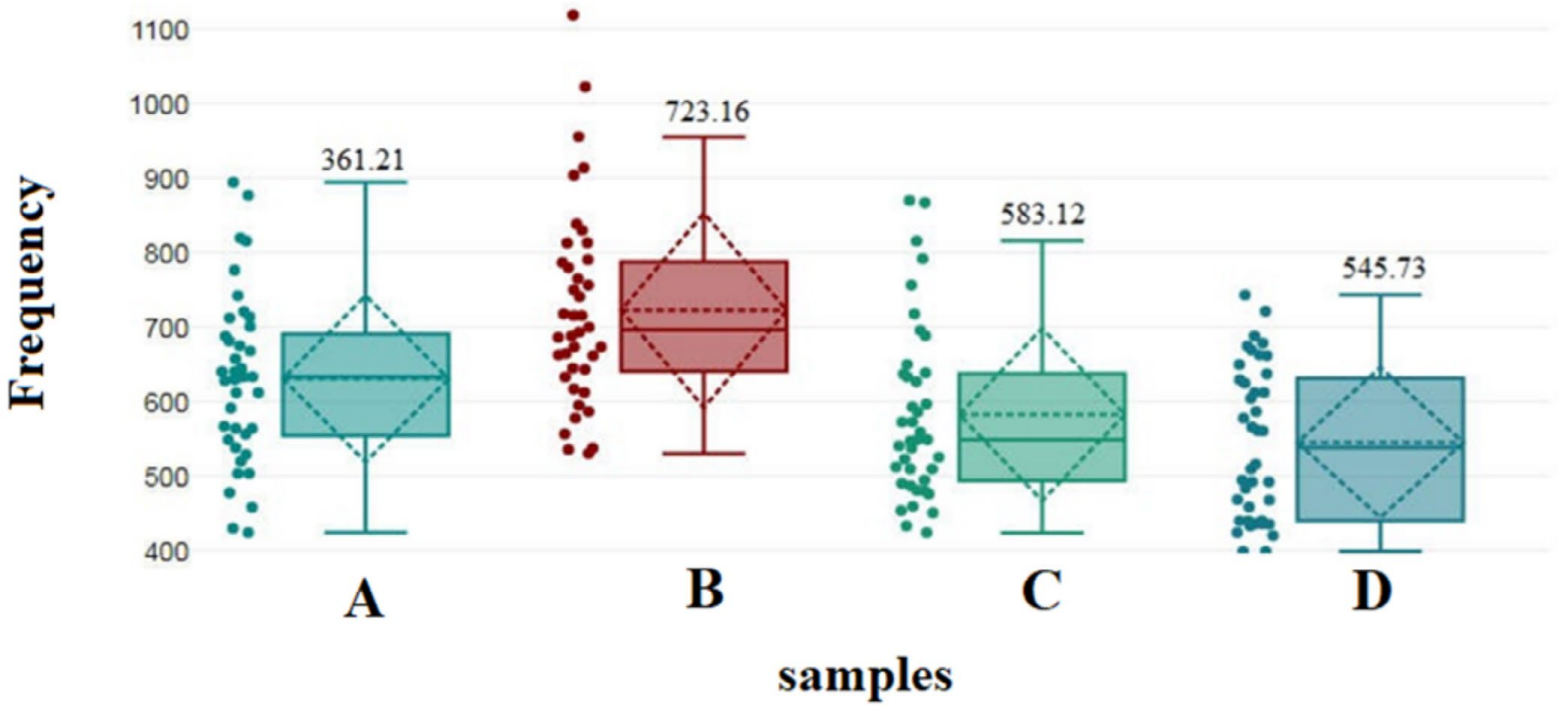
The average fiber diameter in electrospun edible films: Control (A), P‐SE 10% (B), P‐SE 20% (C), and P‐SE 30% (D).

The diameter of the nanofibers in each of the films was measured using ImageJ software (Figure 3). These diagrams show the frequency distribution of fiber diameter in each of the different samples. The diameter frequency of P‐SE 10% was higher, indicating the formation of thicker fibers in this type of film. The data distribution of P‐SE 10% is mainly in the frequency range of 600–800. In the control sample, most of the data falls within the frequency range of 600–700. The data distribution of P‐SE 20% is concentrated in the frequency range of 500–600. However, there are some data of this sample falling outside of this range (up to 1100). P‐SE 30% has a wide distribution, with most of it falling within the frequency range of 500–700 nm.

Figure 3 shows the mean of fiber diameter distribution in four different samples: control, P‐SE 10%, P‐SE 20%, and P‐SE 30%. A box plot provides information about the distribution of data. The middle line in each box represents the median of the data, and the edges of the box represent the first and third quartiles (Q1 and Q3). The ends of the lines show the minimum and maximum data, and each graph represents the data related to electrospun films separately.

The mean data of the control sample is approximately 361.21 nm. For the P‐SE 10% sample, the mean data was approximately 736.16 nm, with a wider distribution of data observed. The mean data of P‐SE 30% was 545.73 nm, and the distribution of data was narrower than that of P‐SE 10%. According to the results of this analysis, it was also proven that P‐SE 10% apparently has larger diameters (higher median) and more dispersion. This difference may be attributed to variations in the manufacturing method or raw materials used. The control sample and P‐SE 30% have a smaller mean diameter than P‐SE 10%, and their dispersion is also lower. The average diameter of sample P‐SE 20% (583.12 nm) is greater than that of the control sample but smaller than that of sample P‐SE 30%.

In a study conducted by Shavisi, the average diameter of nanofibers of electrospun films produced from chitosan‐carrageenan and *Malva sylvestris* anthocyanins was measured and compared using the imageJ software. The morphology of the produced films was reported to be almost uniform, and the fiber diameter of the films containing 4% essential oil was thinner (Shavisi 2024). Tang et al. (2019) investigate the impact of essential oils on gelatin nanofibers used for packaging. Their findings indicate that the addition of essential oils results in an increase in the average diameter of the nanofibers. This change is significant as it affects the mechanical properties and barrier functions of the packaging. The study suggests that maintaining a smooth and uniform morphology is essential for the effective release of encapsulated bioactive compounds, further emphasizing the importance of controlling fiber diameter for optimizing performance in food packaging applications (Tang et al. 2019).

### 3.5 Functional Group Analysis

**FIGURE 4:**
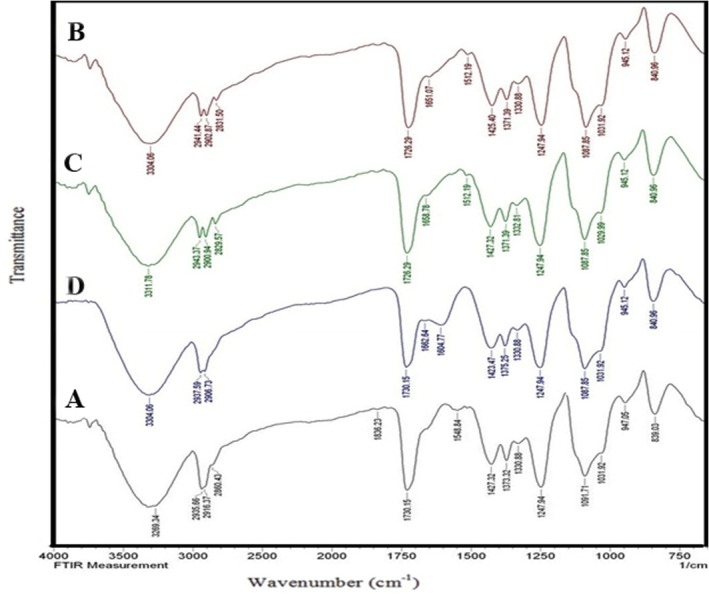
FTIR spectra of electrospun edible films: Control (A), P‐SE 10% (B), P‐SE 20% (C), and P‐SE 30% (D). The input does not contain any text to analyze or correct.

In this research, the functional group was investigated using FT‐IR (Figure 4). The observed peak at 3269 cm^−1^ corresponds to the O‐H stretching vibrations, which are significantly broadened due to hydrogen bonding. The increase in wavenumber across all samples containing *sumac* extract is attributed to the slight reduction and displacement of hydrogen bonds (Forghani et al. 2022).

In all spectra, the peak at 2935 cm^−1^ and its vicinity is a result of C–H stretching vibrations, associated with sp3 hybridization, indicating the absence of double bonds. The sharp and intense peak at 1730 cm^−1^, identifiable in both the control sample and the samples containing sumac extract, corresponds to the C=O stretching vibrations (Jiang et al. 2024).

The peaks at 1427 cm^−1^ and 1373 cm^−1^ are attributed to neutral vibrations in CH_2_ and CH_3_, respectively, present in the PVA structure. The medium to strong peak at 1091 cm^−1^ signifies the C–O stretching and CH–CH2 bending vibrations, confirming the presence of PVA. A new peak of P‐SE 30% emerges at 1604 cm^−1^, indicating the presence of aromatic rings in the sumac sample, which is more pronounced at this specific concentration compared to other concentrations. This peak is, in fact, associated with the C=C stretching vibrations in aromatic rings.

Various studies have similarly interpreted the peaks related to PVA and other compounds, aligning with the results of our study (Yong et al. 2019). In this regard, we can refer to the study conducted by Luo et al. In their research, they prepared electrospun films using tea extract. Through FT‐IR analysis, they investigated the absorption peaks, stretching, bending vibrations, and structure of the film. The study confirmed the presence of polyphenol alcohol in the film's structure (Luo et al. 2020).

In a separate study, anthocyanin and propolis were utilized to create electrospun films using PVA/starch. The FT‐IR spectrum confirmed the presence of anthocyanin and polyvinyl alcohol in the films by analyzing the bonds (Mustafa et al. 2021).

### 3.6 Crystallinity of the Composite Films

**FIGURE 5:**
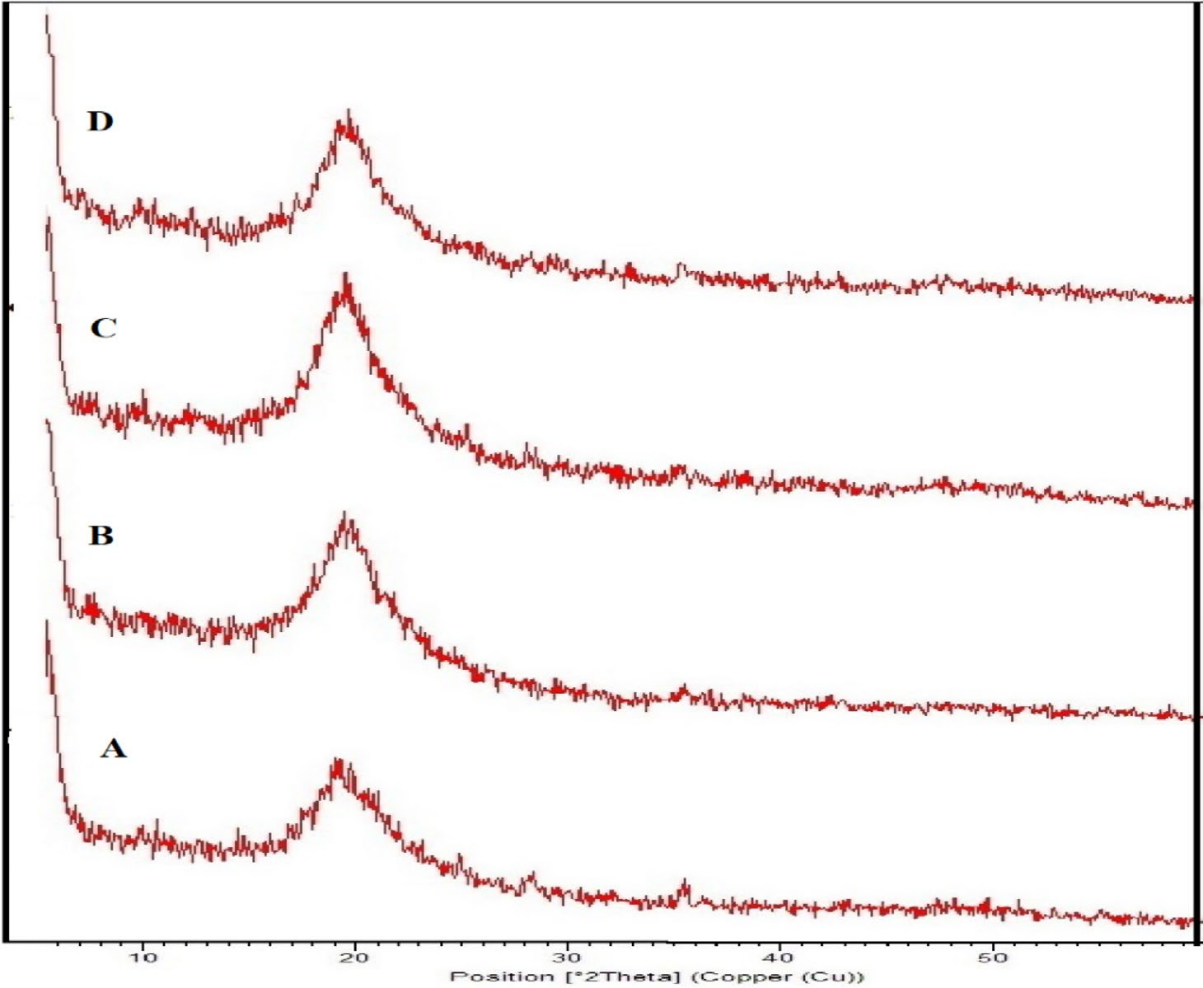
XRD patterns of electrospun edible films: Control (A), P‐SE 10% (B), P‐SE 20% (C), and P‐SE 30% (D).

XRD patterns of electrospun films containing *sumac* extract depicted in Figure 5. All samples exhibit a sharp peak within the range of 2*θ* = 18° to 2*θ* = 22° corresponding to the hydrated crystals in the PVA chain. In a research study conducted by Wen et al., the range of peaks was reported. They used *Nervilia fordii* extract to create electrospun films based on PVA/PVP and showed that the crystallinity index ranged from 19.67° to 24.63° (Wen et al. 2021).

The peak intensity of P‐SE 10%, P‐SE 20%, and P‐SE 30% decreased and became wider compared to the control sample. This state indicates that the crystalline structure is gradually destroyed and the amorphous structure is formed. The high applied voltage may cause the PAN polymer solution to accelerate towards the collector from the needle tip. During this process, the solvent evaporates, the polymer undergoes stretching, and solidifies rapidly into a three‐dimensional structure. As a result, the nanofibers are unable to form a crystalline arrangement (Córdoba and Sobral 2017). Also, in the treatments containing PVA/*Sumac*, the height of the peaks gradually decreased as the sumac concentrations increased, which can be caused by the compatibility of *Sumac* extract to be incorporated in the polymer matrix (Karbownik et al. 2019; Yong et al. 2019).

### 3.7 Tensile Properties and Strength of the Composite Films

Table 2 shows the TS and EAB: TS is a component used to measure the strength of a polymer under the influence of polymer chain interactions in the nanofiber substrate. As it is evident, the samples containing 10% (0.034 MPa) and 20% (0.027 MPa) *Sumac* extract exhibited higher TS compared to the control sample (0.024 MPa). The highest TS was observed in the sample containing 10% *Sumac* extract. The reason for this is the increased interactions of the anthocyanin functional groups in *Sumac*/PVA, leading to a stronger polymer structure. However, in the sample containing 30% (0.024 MPa) *Sumac* extract, the TS was similar to that of the control sample. The EAB decreased with the increasing concentration of the extract, with the lowest value observed in the sample containing 30% sumac extract (74.58%). This can be attributed to the reduced fluidity of the nanofibers and an increase in thickness due to the formation of cross‐links (Ghadiri et al. 2023).

**TABLE 2:** Physical and mechanical properties of electrospun edible films composed of polyvinyl alcohol and sumac extract.

Sample	MC (%)	WS (%)	Opacity (A600/mm)	WVP (g mm/m^2^ Pa)	Thickness (mm)	TS (MPa)	EB (%)
Control	7.14 ± 0.06^a^	30.24 ± 1^a^	39.72 ± 2.07^c^	17.62 ± 0.37^a^	0.013 ± 0.14^c^	0.024 ± 0.0017^c^	250 ± 9.84^a^
P‐SE10%	4.3 ± 0.09^c^	19.08 ± 1.31^c^	175.30 ± 1.09^b^	9.49 ± 0.17^b^	0.061 ± 0.3^a^	0.034 ± 0.002^a^	37.91 ± 7.09^d^
P‐SE20%	5.27 ± 0.02^c^	22.15 ± 1.41^b^	206.22 ± 3.08^a^	9.64 ± 0.17^b^	0.021 ± 0.2^b^	0.027 ± 0.001^b^	50.83 ± 9.32^c^
P‐SE30%	6.17 ± 0.03^b^	26.25 ± 0.73^b^	166.48 ± 3.14^b^	15.51 ± 0.34^a^	0.016 ± 0.15^c^	0.024 ± 0.0017^c^	74.58 ± 14.31^b^

*Note:* Data are shown as mean ± standard deviation (SD). The superscripts different letters in a column indicate significant differences (*P* < 0.05).

Abbreviations: EB, Elongation at brea; MC, Moisture Content; TS, Tensile strength; WS, Water Solubility; Wvp, Water Vapor Permeability.

In general, it can be concluded that the components of the extract used in the film preparation were effectively dispersed within the polymer matrix. This dispersion is linked to the enhancement of the film's mechanical properties. When the extract particles are well dispersed within the structure of the nanofibers, we observe various interactions, such as intermolecular bonds between the nanofibers and the extract particles (Chavoshi et al. 2023; Freitas et al. 2023). In various studies, the mechanical properties of the resulting films were found to depend on the composition of the film, with different results reported (de Barros et al. 2024; Estevez‐Areco et al. 2020; López‐Córdoba et al. 2019; Shavisi 2024). For example, a study can be mentioned in which the results of its mechanical analysis were somewhat contradictory upon our examination. They used sumac extract to prepare electrospun packaging films, and it was proven that with an increase in the concentration of the extract, the tensile strength decreased while the elongation at break increased. It was indicated that this was due to the phenolic compounds present in the extract, which hinder strong plasticization between the polymers used in the preparation of the packaging film (Rezaei et al. 2024).

### 3.8 Thickness Variation and Impact on Composite Film Properties

Research has demonstrated that the thickness of films significantly impacts their barrier properties against moisture, gases, and other environmental factors (Díaz‐Montes and Castro‐Muñoz 2021). Thicker films may provide enhanced protection, but they can also alter the sensory attributes of the food product, creating a need for balance between preservation efficiency and consumer acceptance (Galus et al. 2020).

The thickness of the electrospun films is considered a crucial physical feature in food packaging and can vary depending on the specific application and materials used (Moreira et al. 2020). The electrospinning parameters, such as solution viscosity and applied voltage, play a crucial role in determining the thickness of the resulting nanofibers (He et al. 2021).

In this study, it was found that increasing the concentration of sumac extract resulted in a decrease in thickness. A significant difference in the thickness of P‐SE 10% nanofiber mats compared to the control sample was observed (*p* < 0.05). This was further confirmed by SEM results. The control thickness (0.013 mm), P‐SE 10% (0.061 mm), P‐SE 20% (0.021 mm), and P‐SE 30% (0.016 mm) are reported in Table 2. The thickness of the control sample was lower than the samples containing sumac extract, and the thickness of P‐SE was 30% closer to the control sample. The decreasing trend in thickness observed in samples containing the extract demonstrates its impact on the electrospinning process and successful distribution within the fiber structure. In various studies, *sumac* extract and other plant extracts were used to prepare biodegradable edible and non‐edible electrospun packaging films. In addition to the physicochemical properties, antibacterial, antioxidant, mechanical properties, the thickness of the packaging films was investigated, and different results were reported (Emir et al. 2023; Rosales‐Murillo et al. 2024).

The thickness of electrospun PVA layers is significantly influenced by the natural ingredients added to them, such as essential oils, extracts, and bioactive compounds. This, in turn, affects their performance in food packaging (Lin et al. 2023).

Rezaei et al. utilized various concentrations of sumac extract in creating electrospun packaging films composed of grass pea protein isolate, polyvinyl alcohol, and sumac ( *Rhus coriaria* L.) extract. Their findings mirrored ours, showing a decrease in thickness with higher extract concentrations (Rezaei et al. 2024). However, some studies have indicated that the thickness of electrospun films made with polyvinyl alcohol actually increased when natural bioactive compounds were added. They attributed the increase in viscosity to these compounds, but their results were contradictory to ours (Aminzare et al. 2024).

### 3.9 Light Absorption and Transparency Analysis

The nanofiber mats spun in the present study were opaque. The opacity of the control sample (39.72 A600/mm), P‐SE 10% (206.22 A600/mm), P‐SE 20% (175.30 A600/mm), and P‐SE 30% (166.48 A600/mm) is reported in Table 2. By increasing the concentration of *Sumac* extract to 20%, the opacity of electrospun layers significantly increased. However, in the sample containing 30% *Sumac* extract, the opacity decreased. The reason for this could be that colored particles accumulate at a higher concentration of the *Sumac* extract, leading to a decrease in opacity.

Sun et al. (2020) investigated various physicochemical characteristics, including opacity. They used mulberry anthocyanin extract to prepare an electrospun film. As the concentration of the extract increased, the opacity also increased. This feature was ultimately reported as a positive attribute of the films prepared for food packaging.

### 3.10 Water‐Related Properties of the Composite Films: MC/WS/WVP

According to Table 2, the lowest and highest MC is related to the optimal film with 10% sumac extract and the control sample. By adding sumac extract in different concentrations to PVA, the MC has increased: Control sample (7.14%), P‐SE 10% (4.3%), P‐SE 20% (5.27%), and P‐SE 30% (6.17%). The reduction in MC of food packaging films containing sumac extract is attributed to the interaction between active anthocyanin groups and hydroxyl groups within the molecular structure of the PVA/Sumac extract. Additionally, the diameter of the nanofibers plays a significant role in influencing this behavior following the addition of *Sumac* extract. In 2020, Huang et al. found that incorporating *Arnebia euchroma* root anthocyanins into polyvinyl alcohol films reduced their MC (Huang et al. 2019). Likewise, Zhai et al. (2017) demonstrated that the addition of roselle anthocyanins to starch/polyvinyl alcohol films lowered the MC of the produced films (Zhai et al. 2017).

The solubility of nanofibers in the control sample, P‐SE 10%, P‐SE 20%, and P‐SE 30% was reported as 30.24%, 19.08%, 22.15%, and 26.25%, respectively (Table 2). Among the samples containing sumac extract, the lowest and highest amounts of WS were related to P‐SE 10 and P‐SE 30, respectively.

By adding sumac extract in various concentrations, the solubility significantly decreased compared to the control sample. The reason for this achievement can be attributed to the anthocyanins present in sumac extract, as anthocyanins possess high hydrophilic properties. Actually, Intermolecular interactions between the polymer and bioactive compounds, especially through hydrogen bonding and polar interactions, play a crucial role in reducing the solubility of electrospun films. These interactions lead to the formation of a denser polymeric network that restricts water permeability and prevents swelling and dissolution of the film structure. The presence of phenolic compounds in sumac extract can strengthen hydrogen bonds between PVA chains and reduce the amount of free hydrophilic groups, consequently decreasing the solubility of the film in aqueous environments. Furthermore, the formation of more regular and denser structures as a result of these interactions can improve the mechanical properties and stability of the film, which is highly important in food packaging applications. (Wang et al. 2019).

Ghani et al. (2018) found that increasing the concentration of cinnamon essential oil nanoemulsions reduced the solubility of soy polysaccharide‐based edible films (Ghani et al. 2018). Similarly, Ezati et al. (2019) demonstrated that incorporating anthocyanins from *Lithospermum erythrorhizon* into cellulose papers significantly decreased their water solubility. This effect was linked to the hydrophobic properties of anthocyanins and the polyphenolic compounds they contain (Ezati et al. 2019). Furthermore, Kurek et al. (2018) observed a decline in the solubility of chitosan films as the concentration of blueberry extract increased. They attributed this reduction to the presence of anthocyanins and a corresponding decrease in the availability of free functional groups in chitosan (Kurek et al. 2018).

The WVP of the control sample (17.62 g mm/m^2^ Pa), P‐SE 10% (9.49 g mm/m^2^ Pa), P‐SE 20% (9.64 15.51 g mm/m^2^ Pa), and P‐SE 30% (15.51 g mm/m^2^ Pa) were reported in Table 2. The WVP of samples containing sumac extract was reported to be lower than that of the control sample. The lowest and highest WVP were related to the P‐SE 10 percent sample and the control sample, respectively. WVP is a crucial parameter in the study of pH‐sensitive packaging films. This is because the hydroxyl and carboxyl compounds present in these films, with their hydrophilic composition, significantly hinder WVP (Liu et al. 2022). The reduction process may be attributed to the hydrogen‐covalent interactions between the polymer network and the phenolic compounds found in the sumac extract. These interactions can restrict the availability of hydrogen groups needed to create hydrophilic bonds with water, resulting in a decreased affinity of the film for water (Ardjoum et al. 2023). The SEM images from the current research also revealed that the addition of anthocyanins from sumac extract resulted in a reduction in the diameter of nanofibers, creating a pathway for the passage of steam (Huang et al. 2019). However, according to previous studies, the electrostatic interaction that forms electrospun films reduces their affinity for reacting with water molecules. Therefore, the selection of appropriate polymers and bioactive compounds is important, as WVP is a key factor in packaging films that significantly impacts freshness. This property varies in films made with different polymers. According to the study by Shavisi et al. (2024) adding *Malva sylvestris* extract to the electrospun film based on chitosan‐carrageenan reduces the WVP. The reason for this is the reduction of OH groups, as the fiber mats reacted with the oxygen functional groups present in the extract. This reduced the affinity of the electrospun packaging films for water absorption (Shavisi 2024).

## 4 Conclusions

In this study, electrospun films were prepared using polyvinyl alcohol (PVA) and sumac extract, and their color changes in response to pH variations were investigated. The films exhibited promising antibacterial, antioxidant, physicochemical, structural, and mechanical properties. The sample containing 10% sumac extract was identified as the most suitable in terms of these characteristics, suggesting its potential use in food packaging applications. These films offer a sustainable and effective solution for enhancing food preservation and safety. However, despite their potential, the use of these films in real‐world food packaging faces several challenges that need to be addressed for broader implementation.

One of the key challenges lies in the scalability of the electrospinning process. While electrospinning has been proven to be effective at a laboratory scale, transitioning this process to industrial‐scale production for food packaging can be complex and costly. The scalability of electrospinning for large‐scale food packaging applications needs to be further investigated to ensure that the process can be replicated efficiently without compromising the properties of the films. Additionally, the cost‐effectiveness of using sumac extract compared to other natural colorants and antimicrobial agents is another critical consideration. Although sumac extract offers both antioxidant and antimicrobial benefits, the availability and cost of sumac, as well as the extraction process, may limit its commercial viability in comparison to other bioactive compounds, such as those derived from more widely available sources like rosemary or green tea. Further cost analysis and optimization of the extraction process are required to assess its feasibility for large‐scale production.

Moreover, the stability and durability of the films under real‐world conditions need to be assessed. It is crucial to evaluate whether the films maintain their mechanical and functional properties when exposed to typical storage conditions, such as refrigeration, varying humidity levels, and temperature fluctuations. Studies investigating the long‐term stability and integrity of these films under different environmental factors will be essential in determining their suitability for widespread use. Another important consideration is the impact of these films on the sensory characteristics of the packaged food. Consumer acceptance is largely influenced by the sensory properties of food, including taste, texture, and appearance. The films must not interfere with these attributes, which could limit their acceptability among consumers. It will be necessary to conduct sensory evaluation studies to ensure that the films do not negatively affect the overall quality of the packaged food. Several studies have been conducted on the combination of natural and herbal extracts and essential oils (*cinnamon*, *thyme*, *clove*, and *rosemary* essential oils) with polyvinyl alcohol to produce electrospun packaging films. In some of these studies, electrospun films were investigated as coatings to examine the shelf life of food products (Red meat, poultry, fish, and fruits such as strawberries and apples) and their sensory properties. The results showed that the organoleptic properties were maintained at an acceptable level, ensuring that the product remained completely edible.

In general, while the electrospun films containing sumac extract show great promise as a sustainable food packaging material, several practical limitations must be addressed. Further research is needed to optimize the electrospinning process for industrial‐scale production, reduce costs, and ensure the films maintain their properties under real‐world storage conditions. Additionally, consumer acceptance and the impact on sensory properties must be carefully evaluated to ensure the feasibility of these films in the food packaging industry.

## Author Contributions

**Aylar Shiri:** methodology, software, writing – original draft. **Ehsan Sadeghi** and **Khadije Abdolmaleki:** writing – review and editing, conceptualization, funding acquisition. **Mahya Soltani:** project administration, funding acquisition, writing – review and editing. **Farzad Dabirian, Hooman Shirvani:** formal analysis, investigation, project administration.

## Conflicts of Interest

The authors declare no conflicts of interest.

## Data Availability

The data that support the findings of this study are available on request from the corresponding author.
